# Autoimmune pulmonary alveolar proteinosis successfully treated with lung lavage in an adolescent patient: a case report

**DOI:** 10.1186/s13256-021-02906-2

**Published:** 2021-07-09

**Authors:** Abdalla Mohmed Alasiri, Reem Abdullah Alasbali, Meaad Ali Alaqil, Aishah Marei Alahmari, Nouf Dagash Alshamrani, Rabab Nasir Badri

**Affiliations:** 1 Department of internal medicine, Aseer Central Hospital, Abha, Saudi Arabia; 2 Department of histopathology, Aseer Central Hospital, Abha, Saudi Arabia

**Keywords:** Autoimmune pulmonary alveolar proteinosis, Whole lung lavage, Bronchoalveolar lavage, Crazy-paving pattern, GM-CSF, Case report

## Abstract

**Background:**

Pulmonary alveolar proteinosis is a rare interstitial lung disease characterized by accumulating surfactant materials in the alveoli. The autoimmune form is by far the most common in adults, while in the pediatric age group, the vast majority of cases are congenital. We report a case of an adolescent patient diagnosed with autoimmune pulmonary alveolar proteinosis, which is unusual in this age group.

**Case presentation:**

A-15 year-old Saudi male presented to the emergency department with a history of shortness of breath and low oxygen saturation. High-resolution computed tomography of his chest showed a global crazy-paving pattern. Autoantibodies against granulocyte-macrophage colony-stimulating factor were detected in his serum. A diagnosis of the autoimmune form of pulmonary alveolar proteinosis was confirmed after excluding other possible causes. The patient improved after he underwent whole lung lavage under general anesthesia, and he was independent of oxygen therapy after 6 months of follow-up.

**Conclusion:**

The autoimmune form of pulmonary alveolar proteinosis is rare in the pediatric age group and should be considered when no apparent cause of this disease was found. Whole lung lavage should be the first treatment modality offered in this setting with close follow-up and monitoring.

## Background

Pulmonary alveolar proteinosis (PAP) is a rare disease caused by abnormal accumulation of surfactant material in alveoli [1]. Several forms of PAP exist: primary PAP (either autoimmune or hereditary), secondary PAP due to exposure to a high level of dust (such as silica) or underlying infections or malignancy, and congenital PAP due to defect in the production of surfactant [2, 3].

Primary PAP can be autoimmune (most common) or hereditary [defect in the receptor of colony-stimulating factor 2 receptor alpha and beta (*CSF2RA* and *CSF2RB*)] [4, 5]. Autoimmune PAP (aPAP) develops because of circulating autoantibodies against granulocyte-macrophage colony-stimulating factor (GM-CSF), and most affected individuals present to clinical attention in their fourth or fifth decade of life with progressive shortness of breath (SOB) and productive cough [6, 7].

In this report, we describe a case of a 15-year-old patient diagnosed as a case of aPAP, which is a rare cause of PAP in this age group [8].

## Case presentation

A 15-year-old boy not known to have prior medical illness presented to our hospital emergency department (ED) with a history of shortness of breath upon climbing stairs and blue discoloration of his lips and extremities. There was no history of cough, chest pain, palpitation, fever, or constitutional symptoms. Cyanosis was first noted by his parents on his hands 3 months before the recent presentation. There was no significant exposure to household or environmental fumes, dust, or mineral oils. The father of the patient has rheumatoid arthritis, and his mother has multiple sclerosis. Other aspects of history were unremarkable, including drug history; specifically, the patient was asked about using any immunosuppressant medication.

On physical examination (P/E), the patient was conscious, oriented, and alert but appeared ill and had peripheral and central cyanosis as well as finger clubbing. He was afebrile but tachypneic (respiratory rate 35 breaths per minute), with room air oxygen saturation of 68%, which improved to 92% with 10 L of oxygen therapy delivered through a nonbreathable mask. Chest auscultation revealed diffuse bilateral crackles with a decrease in air entry bilaterally. The rest of the P/E was unremarkable.

**Fig. 1:**
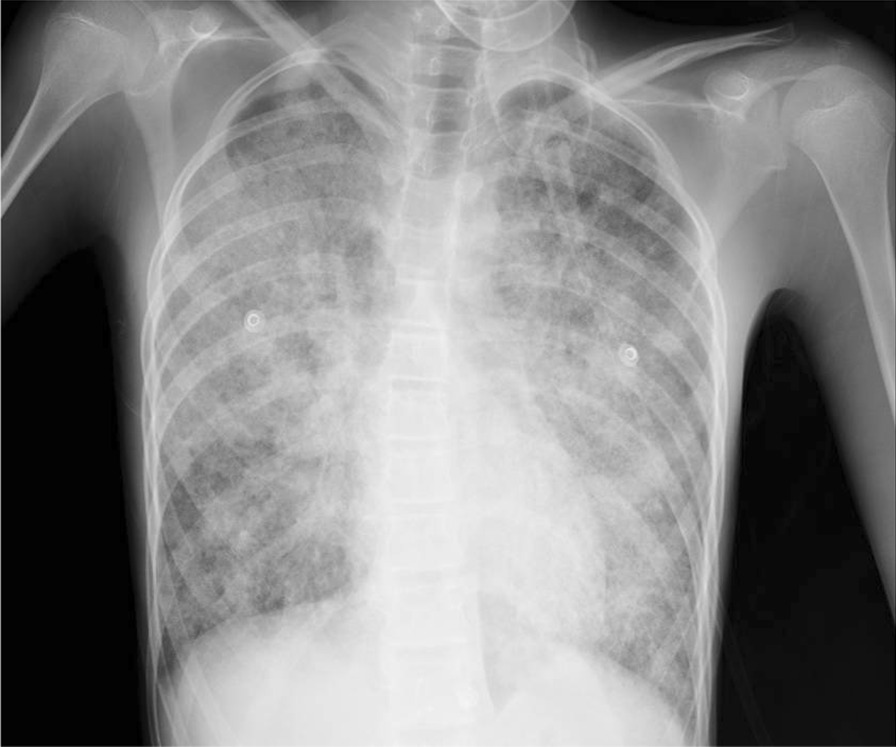
Chest X-ray showing extensive bilateral alveolar scattered and coalescent alveolar infiltrate involving both lung fields

Laboratory investigations in the ED were normal (Table 1) apart from polycythemia and elevated lactate dehydrogenase (LDH). On chest X-ray, extensive bilateral alveolar scattered and coalescent alveolar infiltrates involving both lung fields were evident (Fig. 1). The patient was admitted to the general ward for further evaluation and management. After admission to the general ward, further investigations were conducted, including inflammatory marker and tuberculosis tests, which all returned normal (Table 1).

**Table 1:** Laboratory findings

Inspection item			Reference range
Hematology			
Hematocrit	62.4	%	40–50
Hemoglobin	21.6	g/dL	13–17.5
RBCs	7.53	10^6^/μL	4.5–6.2
WBCs	6.04	10^3^/μL	4–10
Differential counts			
Eosinophils	0.1	10^3^/μL	0.02–0.5
Lymphocytes	3.42	10^3^/μL	1–3
Monocytes	0.64	10^3^/μL	0.2–1
Neutrophils	1.84	10^3^/μL	2–7
Platelets	217	10^3^/μL	150–400
ESR	0	per hour	0–15
PT	11.80	sec	11–16
aPTT	32.16	sec	26–40
INR	1.09	–	0.8–1.3
Biochemistry			
Na	144	mmol/L	135–135
K	4.37	mmol/L	3.5–5.3
Ca	9.01	mg/dL	8.8–10.2
Cr	0.59	mg/dL	0.5–1.3
Urea	15.6	mg/dL	10–50
RBG	89	mg/dL	70–110
ALT	10.5	U/L	00–41
AST	31.4	U/L	00–37
GGT	20	U/L	10–60
ALP	113	IU/L	40–113
Albumin	3.95	g/dL	3.4–4.8
Total protein	6.2	g/dL	6.4–8.3
Total bilirubin	1.12	mg/dL	0–1.10
Direct bilirubin	0.24	mg/dL	0–0.2
LDH	587	U/L	135–225
Serology			
CRP	Negative	mg/dL	0–0.8
RF	Negative	IU/mL	0–15
Serum anti-GM-CSF	Positive	–	Negative
c-ANCA	Negative	–	Negative
p-ANCA	Negative	–	Negative
ACPA	Negative	–	Negative
ANA	Negative	–	Negative
HIV^a^	Negative	–	Negative
HBsAg	Negative	–	Negative
HBsAb	25.1	mIU/mL	10–300
HCV^b^	Negative	–	Negative
Other			
PPD	0	mm	0
BALF culture	Negative	–	Negative
BALF for TB^c^	Negative	–	Negative
PAS stain	Positive	–	Negative

^a^Using fourth-generation antigen and antibodies combination HIV-1/2 immunoassay

^b^Anti-HCV antibodies immunoassay

^c^By acid fast bacilli smear, culture, and polymerase chain reaction

*ACPA* anti-citrullinated peptide antibodies, *ALP* alkaline phosphatase, *ALT* alanine transaminase, *ANA* antinuclear antibodies, *Anti-GM-CSF* anti-granulocyte-macrophage colony-stimulating factor, *aPTT* activated partial thromboplastin time, *AST* aspartate transaminase, *c-ANCA* cytoplasmic antineutrophil cytoplasmic autoantibodies, *Cr* creatinine, *ESR* erythrocyte sedimentation rate, *GGT* gamma-glutamyl transaminase, *HBsAb* hepatitis B surface antibody, *HBsAg* hepatitis B surface antigen, *HCV* hepatitis C virus, *HIV* human immunodeficiency virus; *INR* international normalization rate,* LDH* lactate dehydrogenase, *p-ANCA* perinuclear antineutrophil cytoplasmic antibodies, *PAS stain* periodic acid–Schiff, *PPD* purified protein derivative, *PT* prothrombin time, *RBC* red blood cells, *RBG* random blood glucose, *RF* rheumatoid factor, *WBC* white blood cells

**Fig. 2:**
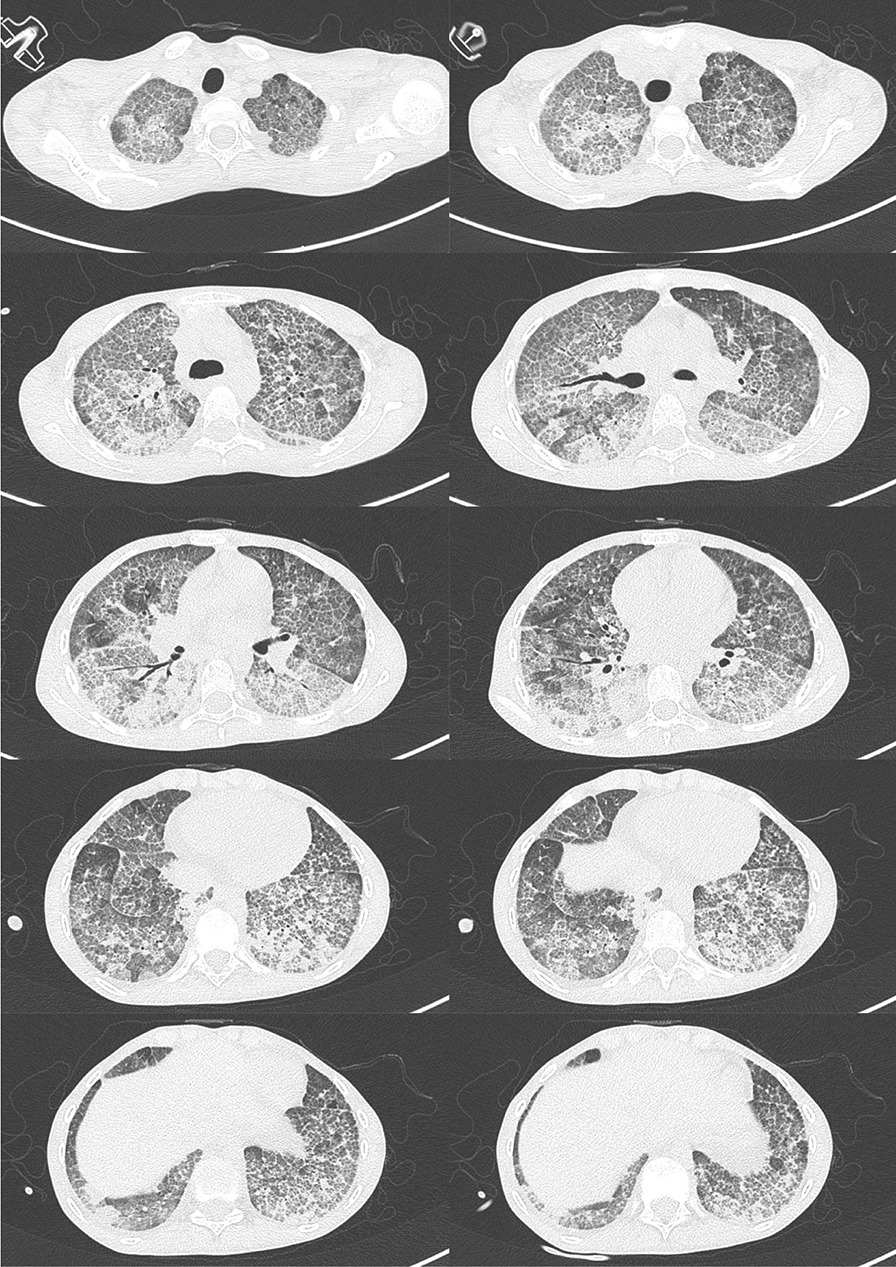
High-resolution tomography of chest showing extensive diffuse bilateral thickening of the lung interstitium with superimposed interlobular septal thickening consistent with “crazy paving” pattern

High-resolution computed tomography (HRCT) of the chest showed extensive diffuse bilateral thickening of the lung interstitium with superimposed interlobular septal thickening and a typical “crazy-paving” appearance (Fig. 2). A pulmonary function test was not done. Collectively, these findings indicate a possible diagnosis of PAP.

**Fig. 3:**
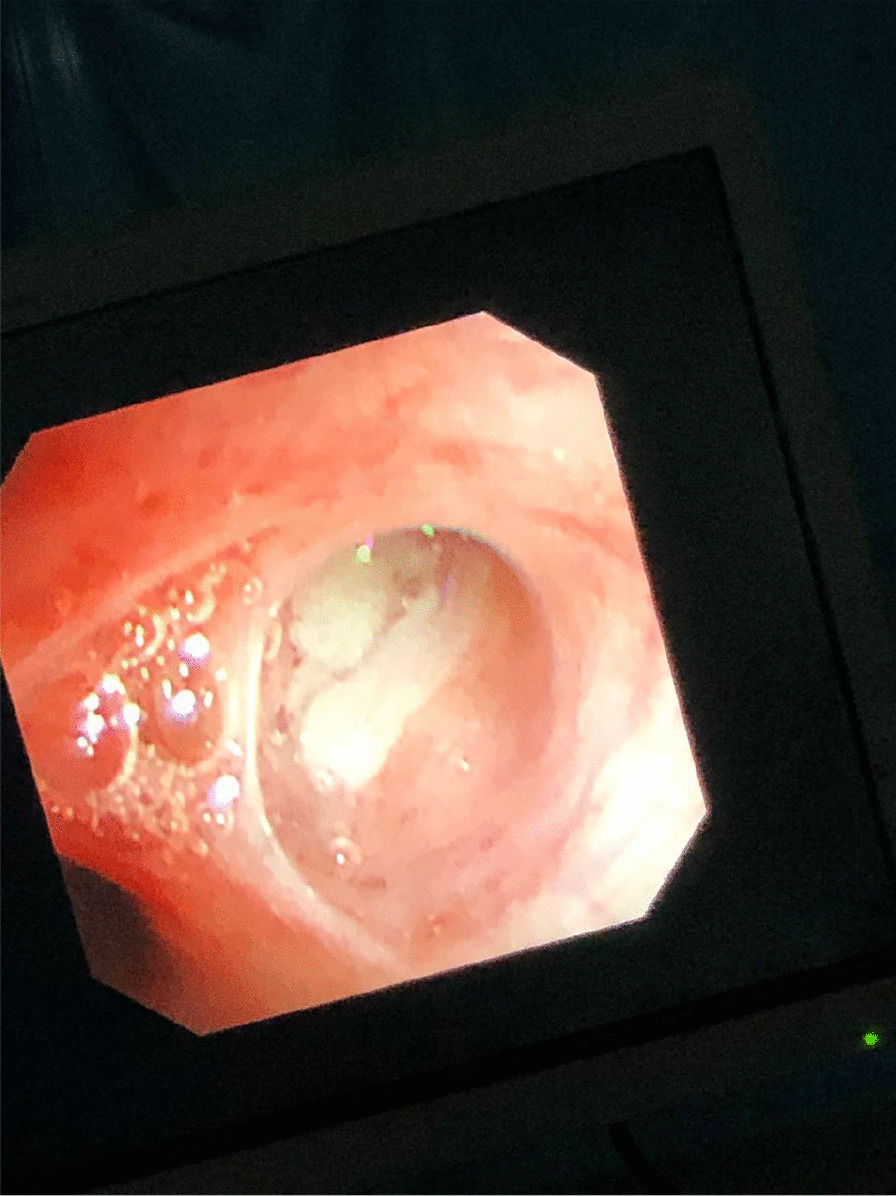
A milky appearance of the secretions throughout all the airway

**Fig. 4.**
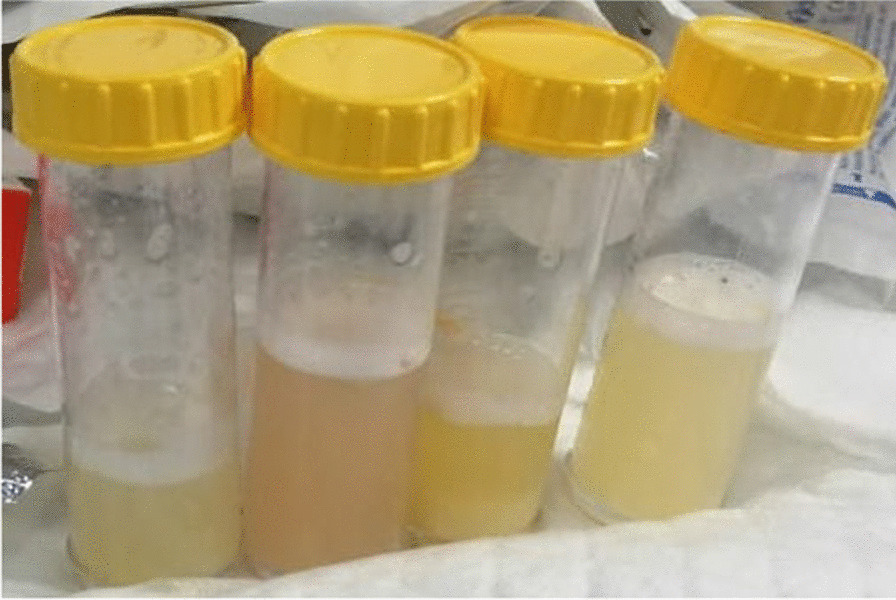
Milky appearance of the bronchoalveolar lavage fluid

The patient then underwent flexible bronchoscopy under general anesthesia, and an apparent milky fluid was aspirated and sent for laboratory testing (Fig. 3). Given the lungs’ abundant secretions, a therapeutic whole lung lavage (WLL) of both lungs was planned for the next day. WLL was done for two separate sessions, starting with the left lung, then followed by the right lung after 3 days, and we did the same procedure steps for both. When the patient arrived at the operating room at the first session, electrocardiographic and invasive arterial pressure monitoring was established. He wore a nonbreathable mask, and oxygen saturation was maintained at >90%. Anesthesia was induced with propofol 25 mg, sevoflurane 2–3%, and fentanyl 100 μg and maintained with sevoflurane 1% infusion. He was intubated with a double-lumen endotracheal tube size 32 Fr, and its correct position was confirmed by fiberoptic bronchoscopy. Oxygen saturation was 100% on intermittent positive pressure ventilation mood with a volume control of 350 mL, positive end-expiratory pressure of 5 cm H_2_O, and airway pressure of 40 cm H_2_O, which were continuously monitored, and regular arterial blood gas analysis. WLL was performed with the patient in the right lateral position on the operating table, and we did sequential lavage with warm saline solution at body temperature followed by passive drainage under gravity. An experienced physiotherapist performed manual chest vibration and percussion. Cycles were repeated until 10 L of total lavage volume was used and clear fluid effluent was obtained (Fig. 4). The procedure lasted approximately 4 hours. The patient was transferred to the intensive care unit for ventilatory support, where he was extubated within 12 hours. Manual chest physiotherapy techniques and positioning maneuvers were continued postoperatively. Finally, a right-sided WLL was planned for within the next 72 hours with the same procedure. After completing the WLL, the patient reported a dramatic improvement in the SOB but still required oxygen therapy.

**Fig. 5:**
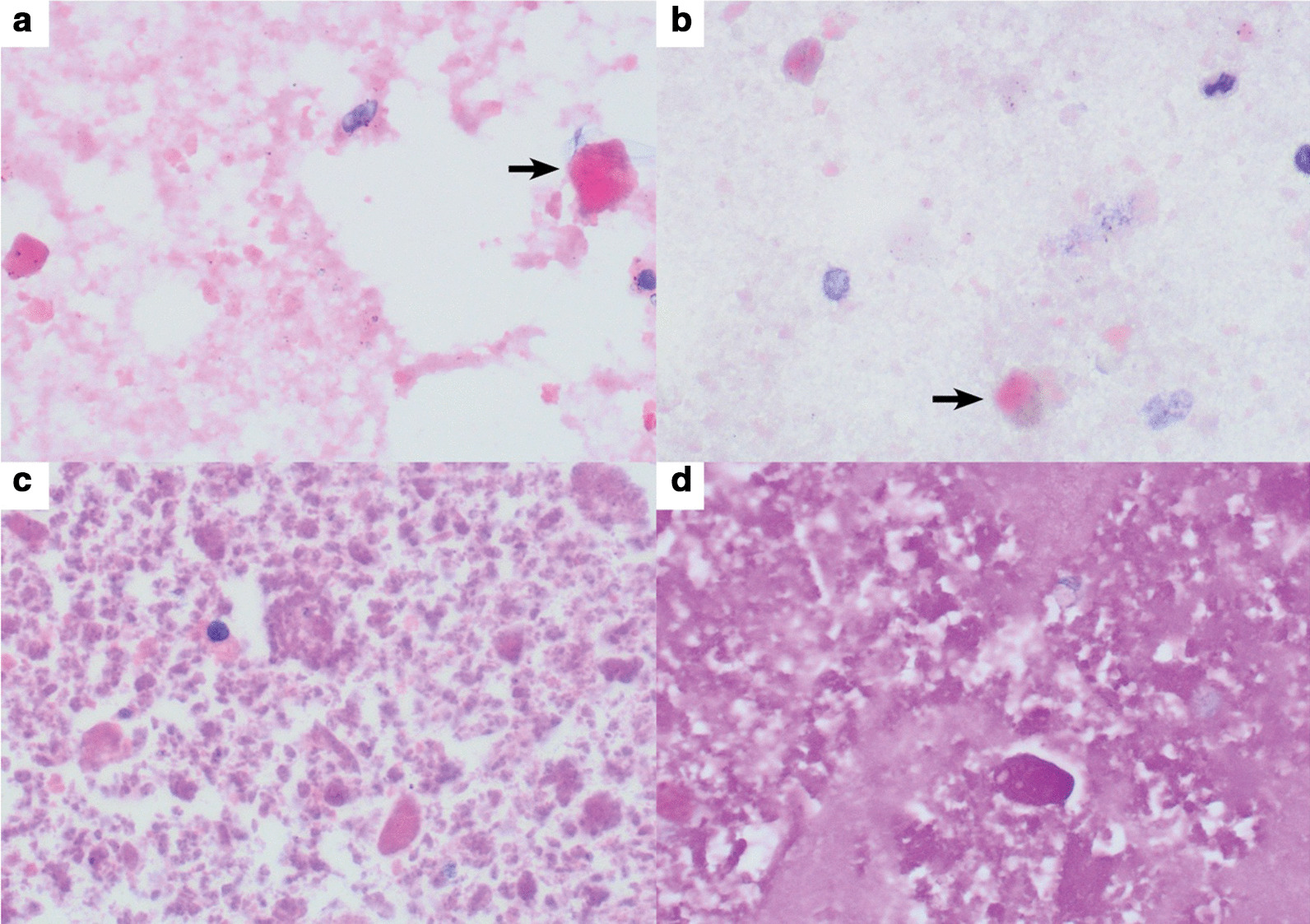
Bronchoalveolar lavage smears showing many dense globules (arrows) in background of finely granular eosinophilic material using (**a**) hematoxylin and eosin stain (H&E) ×400 and (**b**) Papanicolaou stain ×400. Cell block of bronchoalveolar lavage showing granular and globular eosinophilic material that was Periodic acid–Schiff (PAS) positive using (**c**) H&E stain ×400 and (**d**) PAS stain ×400

Cytological examination of the bronchoalveolar lavage fluid (BALF) revealed multiple eosinophilic globules on the background of granular material and inflammatory cells, including macrophages and lymphocytes. They are periodic acid–Schiff (PAS) stain positive (Fig. 5). PAP diagnosis was confirmed, and antibodies against GM-CSF and necessary immunological and infectious workup were requested (Table 1). Antibodies against GM-CSF returned positive in the patient serum. Microbiological investigations of BALF returned negative, including culture studies. Given the absence of clinical and laboratory evidence of hematological, rheumatological, and infectious diseases, and the presence of anti-GM-CSF antibodies in the patient serum and BALF, the diagnosis of aPAP was established. The patient was discharged from the hospital on oxygen therapy, and he maintained oxygen saturation of >93% on 5 L of oxygen for 1 month, then gradually tapered off until he was completely weaned off the oxygen therapy. After 6 months of follow-up, the patient’s oxygen saturation was > 93% on room air, and the symptoms completely disappeared.

## Discussion

Pulmonary alveolar proteinosis is a rare disease first described in 1958 [9]. Since that time, our understanding has improved given advances achieved in the molecular and pathological tools available to clinicians. In contrast to adult PAP, which is usually caused by an autoimmune process [10], most PAP cases in children and adolescents are caused by genetic defects leading to abnormal synthesis of alveolar surfactant [11].

**Fig. 6:**
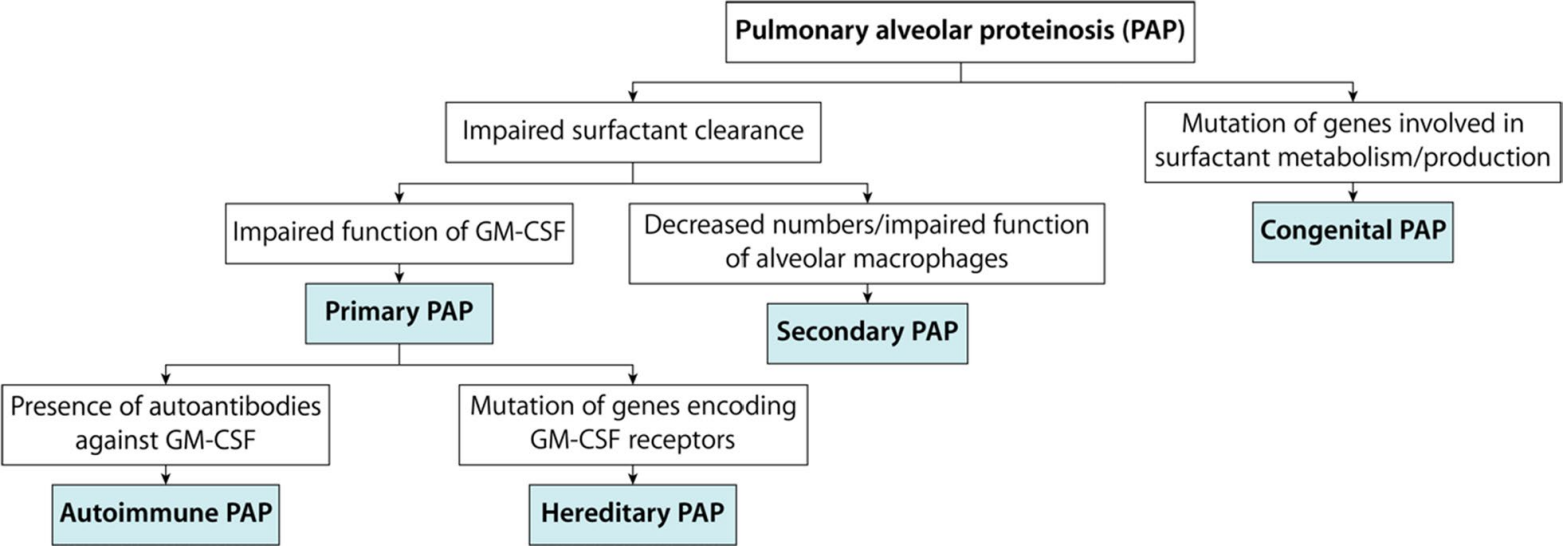
Classification of pulmonary alveolar proteinosis based on underlying cause. *GM-CSF* granulocyte-macrophage colony-stimulating factor

PAP classification is not consistent, and multiple nomenclatures are present for different disease forms [3]. PAP can be categorized into two major categories (Fig. 6). The first category is characterized by impaired surfactant clearance, while the second category’s leading cause is defective surfactant metabolism/production.

The disorders caused by impaired surfactant clearance account for most PAP cases in adults [12]. Impaired surfactant clearance manifests because of either diminished GM-CSF function, also known as primary PAP, or decreased number/impaired function of alveolar macrophage, also known as secondary PAP [13]. Primary PAP is further classified into autoimmune and hereditary. Autoimmune PAP is the most common form of PAP in adults and is characterized by circulating autoantibodies against GM-CSF in the patient’s serum and/or BALF [4, 6]. Hereditary PAP is caused by defects in genes encoding GM-CSF receptors [13]. Infection, malignancy, immunodeficiencies, rheumatological diseases, or inhalation of chemicals or minerals can lead to secondary PAP [6, 14].

GATA binding protein 2 is a transcription factor that is encoded by the *GATA2* gene. It is essential for normal hematopoiesis [15]. Haploinsufficiency *GATA2* gene causes deficiency in GATA binding protein 2, which has heterogeneous presentation including hematologic disorders (such as familial myelodysplastic syndrome/acute myeloid leukemia), immunodeficiency, secondary PAP, and dermatologic disorders (such as panniculitis and erythema nodosum) [16–19]. We exclude the possibility of *GATA2* mutation in our patient as a cause of PAP for two reasons. First, the clinical history (including family history) and laboratory findings were not consistent with the clinical presentation of *GATA2* gene mutation. However, we cannot exclude *GATA2* mutation as some patients may be initially asymptomatic and have completely normal laboratory studies [20, 21]. Second, the patient has autoantibodies in his serum against GM-CSF, which has an accuracy of almost 100% [22, 23]. Taken collectively, the patient’s clinical presentation and laboratory investigations support the diagnosis of primary PAP (with aPAP subtype), not secondary PAP.

Autoimmune PAP is rarely reported in children and adolescents. It may be underdiagnosed given the rarity of the disease and because the test of anti-GM-CSF is expensive and not widely available [10]. We conducted a PubMed search using the following terms: “pulmonary alveolar proteinosis” in combination with “children,” “adolescent,” and “pediatric.” We found 13 cases of pediatric aPAP reported in the literature (Table 2). We cannot exclude the possibility of publication bias. At the onset of the symptoms, the mean age of patients was 12 (±3.04) years; the majority of them were female (64.28%) [24–36].

**Table 2.** Summary of autoimmune pulmonary alveolar proteinosis cases reported in children and adolescents

Case	Age (years)^a^	Gender	Presenting symptoms	Chronic diseases	Treatment	Outcome	Country
Latzin *et al*. [36]	11^b^	M	Exertional SOB	NR	WLL	Partially improved	France
Price *et al*. [35]	13	M	Mild exertional SOB Productive cough FTT	No	WLL Inhaled rhGM-CSF	Improved	Canada
Yamamoto *et al*. [34]	9	F	Dry cough	No	WLL Inhaled rhGM-CSF	Improved	Japan
Robinson *et al*. [33]	16	F	Mild SOB	No	WLL Inhaled rhGM-CSF	Improved	USA
DiBlasi *et al*. [32]	13	M	SOB Cough Fatigue Weight loss	NR	WLL	Improved	USA
Strickler *et al*. [31]	13	F	SOB Dry cough Fever Exercise intolerance Fatigue	No	WLL	Improved	Chile
Sideris *et al*. [30]	6	F	NR	Niemann–Pick disease	WLL Inhaled rhGM-CSF	Improved	USA
Trukalj *et al*. [29]	10	M	Frequent respiratory infections Cough	No	WLL Inhaled rhGM-CSF Bilateral lung transplantation	Improved	Croatia
Gajewska *et al*. [28]	14	F	Exertional SOB Cough	No	WLL Inhaled rhGM-CSF	Improved	Denmark
Sirin *et al*. [27]	15	F	Exertional SOB	No	WLL Inhaled rhGM-CSF	Improved	Turkey
Feld *et al*. [26]	16	F	Exertional SOB Cough Weight loss	Raynaud syndrome	WLL Inhaled rhGM-CSF	Improved	USA
Meka *et al*. [25]	17	F	Exertional SOB Cough	No	WLL	NR	USA
Shivji *et al*. [24]	13	F	Exertional SOB	No	WLL	Improved	Canada
Current report	15	M	Exertional SOB Dry cough	No	WLL	Improved	Saudi Arabia

^a^Age of patient at onset of symptoms

^b^Disease onset was at age of 11 years. However, the bronchoalveolar lavage fluid was obtained and analyzed at age of 24 years with evidence of elevated level of anti-GM-CSF

*F* female, *FFT* failure to thrive, *M* male, *NR* not reported, *rhGM-CSF* recombinant human granulocyte-macrophage colony-stimulating factor, *SOB* shortness of breath, *WLL* whole lung lavage

The clinical presentation of aPAP typically follows an insidious and progressive course [37]. Among the reported cases of pediatric aPAP, the following symptoms were reported (Table 2): exertional shortness of breath (78%), cough (dry or productive) (64%), fatigue (14%), fever, weight loss, failure to thrive, and recurrent respiratory infections (7% each) [24–36]. Lung crackles, finger clubbing, cyanosis, and respiratory distress may be noted upon clinical examination [7]. There are no characteristics laboratory findings for the diagnosis of aPAP except an elevated level of anti-GM-CSF [2]. The reported sensitivity and specificity of anti-GM-CSF in diagnosing aPAP is 100% [22, 23]. However, it is expensive and not widely available. Our patient had polycythemia secondary to chronic hypoxemia and mildly elevated LDH, which has been reported for some patients [38, 39]. Radiological findings are not specific for aPAP but can help narrow the possible diagnosis [40, 41]. On chest X-ray (CXR), there are bilateral patchy infiltrates with or without bronchogram, and on HRCT, there is a crazy-paving pattern (ground-glass opacities superimposed on septal thickening), which is reported to be more common in aPAP than in other forms of PAP [42–44]. Diagnosis of aPAP is suspected when typical radiological findings are present in HRCT along with supportive clinical presentation and exclusion of possible secondary causes that can cause PAP [2, 37]. A definitive diagnosis of aPAP required the presence of typical histopathological findings from a lung biopsy or BALF and detection of anti-GM-CSF antibodies in patients’ serum and/or BLAF [2, 10]. A stepwise approach for diagnosing aPAP has been suggested by some experts [10].

Management of aPAP depends on the clinical status of the patient. Asymptomatic patients may be closely observed and monitored without specific treatment [37]. For symptomatic patients, WLL is the most effective form of treatment and, for a long time, considered the “standard of care” [45, 46]. Data suggest that the introduction of the WLL to the treatment plan of PAP significantly improved 5-year survival [45]. After WLL, patients usually show a remarkable improvement in their clinical and functional status [47, 48]. No drug has been approved for the treatment of aPAP in any country [49]. Given the GM-CSF dysfunction due to neutralizing autoantibodies, efforts were made to address this pathological process by administering recombinant GM-CSF (rhGM-CSF) [50]. Nevertheless, this approach is off-label, expensive, not widely available, and less effective than WLL when used as monotherapy [49, 51, 52]. On account of these limitations, rhGM-CSF therapy is mostly used as augmentation therapy for patients with unsatisfactory responses to first-line treatment [2, 53]. The efficacy of rhGM-CSF depends on the route of administration, dose, and duration of therapy. A 2018 meta-analysis showed that nebulized rhGM-CSF is more effective than subcutaneous rhGM-CSF [54]. A subsequent randomized controlled trial (RCT) has confirmed this conclusion and further supports the role of inhaled rhGM-CSF as adjustment treatment to WLL for aPAP [49, 51]. The response rate appeared to increase with a continuous and prolonged course of treatment proportionally [49, 51, 55]. It is important to mention that we are not aware of randomized trials of rhGM-CSF therapy in children or adolescents. Trials of rhGM-CSF excluded this age group; hence, there are limited data on this therapy’s safety and efficacy in the pediatric age group and adolescents [26–30, 33–35].

The optimum salvage treatment for refractory cases in the pediatric age group and adolescents is unknown. Targeting autoantibodies with rituximab and plasmapheresis have been used with variable success in adult patients [56–59]. Lung transplantation is a more aggressive and invasive intervention and considered the last-resort option. There is one case report of successful treatment of aPAP in a 4-year-old child who underwent bilateral lung transplantation after failure of inhaled rhGM-CSF (Table 2) [29]. However, the patient developed monomorphic post-transplant lymphoproliferative disease (PTLD) associated with Epstein–Barr virus infection and posterior reversible encephalopathy syndrome (PRES). More studies are needed to elucidate the exact role of these invasive and aggressive interventions in treating children and adolescents with aPAP. Corticosteroids have a long history of safety and efficacy in treating multiple autoimmune diseases, and it may seem conceivable that they are useful in aPAP treatment. However, limited data refuted this proposal and showed the contrary by increasing morbidity and mortality [60]. Hence, they have no role in the treatment of aPAP.

## Conclusion

PAP is a rare interstitial lung disease with multiple types and clinical presentations. aPAP is not the usual form in children and adolescents. However, it should be considered in the differential diagnosis after excluding more common causes such as congenital and secondary forms. WLL should be the first-line treatment with or without inhaled rhGM-CSF.

## Data Availability

Data are available from the corresponding author upon reasonable request.

## Abbreviations

ACPA: Anti-citrullinated peptide antibodies
ALP: Alkaline phosphatase
ALT: Alanine transaminase
ANA: Antinuclear antibodies
Anti-GM-CSF: Anti-granulocyte-macrophage colony-stimulating factor
aPAP: Autoimmune pulmonary alveolar proteinosis
aPTT: Activated partial thromboplastin time
AST: Aspartate transaminase
BALF: Bronchoalveolar lavage fluid
c-ANCA: Cytoplasmic antineutrophil cytoplasmic autoantibodies
Cr: Creatinine
ED: Emergency department
ESR: Erythrocyte sedimentation rate
FFT: Failure to thrive
GGT: Gamma-glutamyl transaminase
GM-CSF: Granulocyte-macrophage colony-stimulating factor
H&E: Hematoxylin and eosin stain
HBsAb: Hepatitis B surface antibody
HBsAg: Hepatitis B surface antigen
HCV: Hepatitis C virus
HIV: Human immunodeficiency virus
INR: International normalization rate
LDH: Lactate dehydrogenase
NR: Not reported
p-ANCA: Perinuclear antineutrophil cytoplasmic antibodies
P/E: Physical examination
PAP: Pulmonary alveolar proteinosis
PAS: Periodic acid–Schiff stain
PPD: Purified protein derivative
PT: Prothrombin time
RBC: Red blood cells
RBG: Random blood glucose
RF: Rheumatoid factor
rhGM-CSF: Recombinant human granulocyte-macrophage colony-stimulating factor
SOB: Shortness of breath
WBC: White blood cells
WLL: Whole lung lavage
